# Oxidative Stress Enhances Neurodegeneration Markers Induced by Herpes Simplex Virus Type 1 Infection in Human Neuroblastoma Cells

**DOI:** 10.1371/journal.pone.0075842

**Published:** 2013-10-04

**Authors:** Soraya Santana, Isabel Sastre, Maria Recuero, Maria J. Bullido, Jesus Aldudo

**Affiliations:** 1 Centro de Biología Molecular “Severo Ochoa”, CBMSO (UAM/CSIC), Madrid, Spain; 2 Centro de Investigación Biomédica en Red sobre Enfermedades Neurodegenerativas (CIBERNED), Madrid, Spain; Cincinnati Childrens Hospital Medical Center, United States of America

## Abstract

Mounting evidence suggests that Herpes simplex virus type 1 (HSV-1) is involved in the pathogenesis of Alzheimer’s disease (AD). Previous work from our laboratory has shown HSV-1 infection to induce the most important pathological hallmarks of AD brains. Oxidative damage is one of the earliest events of AD and is thought to play a crucial role in the onset and development of the disease. Indeed, many studies show the biomarkers of oxidative stress to be elevated in AD brains. In the present work the combined effects of HSV-1 infection and oxidative stress on Aβ levels and autophagy (neurodegeneration markers characteristic of AD) were investigated. Oxidative stress significantly potentiated the accumulation of intracellular Aβ mediated by HSV-1 infection, and further inhibited its secretion to the extracellular medium. It also triggered the accumulation of autophagic compartments without increasing the degradation of long-lived proteins, and enhanced the inhibition of the autophagic flux induced by HSV-1. These effects of oxidative stress were not due to enhanced virus replication. Together, these results suggest that HSV-1 infection and oxidative damage interact to promote the neurodegeneration events seen in AD.

## Introduction

Most of the human population is infected with Herpes simplex virus type 1 (HSV-1), which causes life-long latent infections in neurons. Different stimuli induce HSV-1 reactivation, which usually leads to little more than renewed fever blisters. However, on some occasions, new viral particles may spread within the central nervous system, causing encephalitis, meningitis and even epilepsy [1].

HSV-1 infection has also been associated with sporadic Alzheimer’s disease (AD). The first evidence for this emerged when epidemiological studies showed that people infected with HSV-1 who also carried the apolipoprotein E type 4 allele were at higher risk of developing the disease [2]. Other studies have now connected HSV-1 with the main neuropathological hallmarks of AD. For example, HSV-1 is now known to induce the accumulation of β-amyloid peptide (Aβ) [3],[4],[5],[6], hyperphosphorylated tau protein [7], [8], [9], [10] and immature autophagic vesicles [11], [12] in several infection models. Recently, the presence of IgM anti-HSV antibodies in serum - a marker of recent HSV reactivation - was also correlated with an increased risk of developing AD [13]. In addition, the analysis of data gathered in genome-wide association studies involving thousands of Europeans with AD and controls [14] identified a set of AD-linked gene variants that might increase the susceptibility of the brain to HSV-1 infection [15].

A growing number of studies also point to oxidative stress as key in the pathogenesis of neurodegenerative diseases. The brain is particularly susceptible to oxidative stress given its high polyunsaturated fatty acid content, high oxygen demand, and low levels of antioxidants [16]. An increase in markers of oxidative stress in AD brains, including protein, RNA and DNA damage and lipid peroxidation, has been reported, and experimental data from AD animal models confirm the presence of oxidative stress during early disease development [17]. In addition, oxidative stress plays a prominent role in the progression of AD and contributes towards the generation of Aβ deposits and neurofibrillary tangles (reviewed in [18]). However, the oxidative stress hypothesis has recently come under fire, largely due to the negative results obtained in clinical trials with antioxidants [19].

Herpesvirus infections are frequently associated with the generation of oxidative stress in infected cells. HSV-1 has been reported to induce the depletion of glutathione, the main antioxidant defence [20], [21], and to increase ROS levels and lipid peroxidation [22]. In addition, numerous studies have shown oxidative damage to occur in different cell and animal models of HSV-1 infection (reviewed in [23], [24]).

The present work examines the interaction between oxidative stress and HSV-1 infection in the appearance of neurodegeneration markers characteristic of AD. Both mild oxidative stress and HSV-1 infection impaired the autophagic process and inhibited Aβ secretion. In addition, oxidative stress significantly enhanced the effects of HSV-1 on Aβ accumulation and secretion, as well as the impairment of autophagy. These effects are not mediated by the facilitation of infection since oxidative stress reduced the quantity of viral DNA and proteins present and the formation of viral infective particles in HSV-1-infected cells. The results therefore suggest that the increase in oxidative stress concomitant with aging promotes the neurodegeneration events associated with HSV-1 infection.

## Materials and Methods

### Drugs, Plasmids and Antibodies

The rapamycin (0.2 µg/ml), xanthine (10 µM) and bafilomycin A1 (100 nM) used in this study were obtained from Sigma. Leupeptin (0.1 mM) and xanthine oxidase (50 mU/ml) were obtained from Roche. 4′, 6-diamidino-2-phenylindole (DAPI; 5 µg/ml) and ammonium chloride (20 mM) were purchased from Merck.

The GFP-LC3 expression vector (pGFP-LC3) and the mCherry-GFP-LC3 construct (dtLC3) were kindly provided by T. Yoshimori and N. Mizushima [25] and by T. Johansen [26] respectively. Goat anti-MAP LC3 (F14) was supplied by Santa Cruz Biotech. Antibodies that recognized HSV-1 proteins were supplied by DAKO (rabbit polyclonal anti-HSV-1), Sigma (rabbit polyclonal anti-VP16) and Abcam [anti-HSV1 gC Envelope Protein [3G9] and anti-HSV 1 ICP4 Immediate Early Protein (10F1)]. Rabbit anti-GFP serum, rabbit polyclonal anti-Aβ40 and rabbit polyclonal anti-Aβ42 unconjugated antibodies were purchased from Invitrogen. Mouse monoclonal anti-tubulin antibody was supplied by Sigma. The secondary antibodies used for immunostaining were horseradish peroxidase-coupled and purchased from VECTOR, or labelled with Alexa Fluor 488 or 555 dye and purchased from Invitrogen.

### Cell Cultures and Transfection

SK-N-MC human neuroblastoma cells were obtained from the American Type Culture Collection (HTB10). Cells stably expressing human APP (SK-APP, cell line C2) and rat LC3 fused to EGFP (SK-LC3) have been described elsewhere [5], [27]. All SK-N-MC cells were grown as monolayers in minimal Eagle’s medium (MEM) supplemented with 10% heat-inactivated foetal calf serum (FCS), 2 mM glutamine and 50 µg/ml gentamicin. Vero cells were passaged in Dulbecco’s modified Eagle medium (DMEM) supplemented with 5% FCS, 2 mM glutamine and 50 µg/ml gentamicin. All cells were grown at 37°C in a 5% CO_2_ atmosphere. For dtLC3 cell assays, SK-N-MC cells were transiently transfected using the Amaxa® cell line Nucleofector® method following the manufacturer’s instructions. Briefly, a pellet of 4×10^6^ cells was resuspended in 100 µl of Nucleofector® solution V, mixed with 15 µg of dtLC3 plasmid, and subjected to nucleofection following the program C-009. After electroporation, the cells were seeded onto the growth medium. Two days after transfection, they were treated with the appropriate stimuli.

### Infection Conditions and Exposure to Oxidative Stress

The wild-type HSV-1 strain KOS 1.1 was propagated and purified as previously described [28]. SK-N-MC cells seeded in complete MEM at 70–80% confluency were exposed to HSV-1 at 37°C for 1h. Mock infections were performed using a virus-free suspension. Unbound virus was removed and the cells incubated in complete MEM at 37°C. Time and multiplicity of infection (moi; expressed as plaque-forming units [pfu] per millilitre) conditions are indicated in each experiment. The particle to PFU ratio was not determined. The infectious titres of HSV-1 were determined by plaque assay. Briefly, the titration of serially diluted HSV-1 was performed in Vero cells grown in 24-well plates. Cells were overlain with a mixture of DMEM containing 2% FCS and 0.7% agar. After 48 h the cells were fixed and stained overnight with 1% crystal violet in 5% formaldehyde and the plaques counted.

To induce oxidative stress, SK-N-MC cells were placed in fresh medium for 1 h before X-XOD addition. Exposure times are indicated in each experiment. In samples exposed to oxidative stress and HSV-1, X-XOD was added during the time of virus adsorption and maintained until the end of infection.

### Measurement of Secreted and Intracellular Aβ

For ELISA assays, SK-APP cells were mock-infected or infected with HSV-1 and the conditioned media and cell lysates assayed for human Aβ40 and Aβ42 using commercial ELISA sandwich kits (Invitrogen) according to the manufacturer’s instructions. Briefly, the conditioned media were treated with Complete Mini Protease Inhibitor Cocktail (Roche) and concentrated 10-fold by lyophilization. The cells were then suspended in lysis buffer (0.5% Triton X-100, 2.5 mM EDTA in PBS containing the same protease inhibitor cocktail) and sonicated for 60 s. Media and lysate samples were added to ELISA plates pre-coated with mouse monoclonal antibodies specific for the N-terminus of human Aβ (the capture antibody), followed by a rabbit anti-mouse antibody specific for the amino acid sequence (amino acids 1–40 or 1–42) of Aβ (the detection antibody). The detection antibody bound was quantified using a horseradish peroxidase-labelled anti-rabbit antibody producing a coloured signal. The absorbance was read at 450 nm within 30 min of the completion of the procedure. Comparison with the Aβ standard curve allowed the calculation of absolute values for Aβ40 and Aβ42 (in pg/ml of incubation medium, or pg/mg of protein).

### Immunofluorescence Imaging

Immunofluorescence assays were performed on cells grown on coverslips. These were fixed in 4% paraformaldehyde and incubated with the appropriate primary and secondary antibodies. DAPI (5 µg/ml) was added 10 min before the end of the procedure to visualize the nuclei. GFP-LC3 and dtLC3 imaging was performed in the same way but without antibody incubations. All cells were examined using a Zeiss LSM510 META confocal microscope or a Zeiss Axiovert 200 fluorescence microscope equipped with 63× and 100× oil-immersion objectives. Images were captured by a Spot RT slider digital camera (Diagnostic) using MetaMorph™ imaging software, and processed using Adobe Photoshop CS4. Fluorescent-tagged dtLC3 spots were counted and processed using Image J software (NIH).

### Immunoblot Analysis

For immunoblot assays, cells were lysed in cell lysis buffer (50 mM Tris-HCl pH 7.6, 300 mM NaCl and 0.5% Triton X-100) containing Complete Mini Protease Inhibitor Cocktail (Roche), and incubated for 30 min at 4°C. The protein concentration of the lysates was quantified using the BCA Kit (Pierce). Cell lysates were mixed with 2× Laemmli buffer, sonicated, and heated for 5 min at 100°C. After electrophoretic separation, the gels were blotted and stained with the appropriate antibodies. A peroxidase-coupled antibody was used as the secondary antibody. Detection by enhanced chemiluminiscence was performed using ECL™ Western Blotting Detection Reagents (Amersham Biosciences) according to the manufacturer’s instructions.

### Analysis of Cell Viability

Cell viability was assessed using the 3-(4,5-dimethylthiazolyl-2)-2,5-diphenyl tetrazolium bromide (MTT; Sigma) assay. Briefly, M-96 plates were seeded at a rate of 5×10^5^ cells/well and, after exposure to the different stimuli, incubated with 0.5 mg/ml MTT for 3 h at 37°C. The MTT/formazan released from the cells during overnight incubation at 37°C with 100 µl extraction buffer (20% sodium dodecyl sulphate [SDS], 50% formamide adjusted to pH 4.7 with 0.02% acetic acid and 0.025 N HCl) was then determined. Optical densities were measured at 570 nm using an automated Model 680 (Bio-Rad) microplate reader.

### Long-lived Protein Degradation

SK-N-MC cells were labelled for 24 h in medium containing 60 µM unlabelled leucine and [^3^H]leucine (1 µCi/ml). They were then washed and incubated for 24 h in MEM containing 2 mM unlabelled leucine. After the chase, cells were incubated in MEM plus rapamycin, or treated with X-XOD and infected with HSV-1 for 18 h. The cells and media were then collected and separately precipitated by the addition of 10% ice-cold trichloroacetic acid (TCA). Radioactivity was then measured in a liquid scintillation counter. The percentage of long-lived protein degradation was calculated as (TCA-soluble counts from medium)/(TCA-soluble counts from medium)+(TCA-insoluble counts from the cells) [29].

### HSV-1 DNA Quantification

The concentration of HSV-1 DNA was quantified by real-time quantitative PCR as previously described [30] using the Custom TaqMan assay (a specific assay for a sequence belonging to the US12 viral gene), employing an ABI Prism 7900HT SD system (Applied Biosystems) The quantification of human genomic DNA was performed using an Assay-On-Demand probe specific for the GAPDH housekeeping gene (Applied Byosystems; item n° Hs99999905_m1). The quantification results were calculated as viral DNA copy numbers per ng of genomic DNA.

### Statistical Analysis

Bar graph values are expressed as means and standard errors of the mean (SEM). Differences between groups were analysed using the Student t-test. Significance was recorded at p<0.05 (*), 0.01 (**) and 0.001 (***). Before analysis, the largest and the smallest variances were tested for homogeneity using the F-test.

## Results

### Oxidative Stress Enhances the Accumulation of Intracellular Aβ and the Inhibition of Aβ Secretion Induced by HSV-1 Infection

We have previously shown that HSV-1 causes the accumulation of intracellular Aβ and intensely inhibits its secretion [5]. In addition, we reported that, in SK-N-MC cells, the xanthine/xanthine oxidase (X-XOD) free radical generating system regulates the metabolism/processing of the APP protein [31], [32]. Since both events are putative primary events in AD pathogenesis, the effects of oxidative stress on the neurodegeneration markers induced by HSV-1 infection in neuronal cell models were examined.

**Figure 1:**
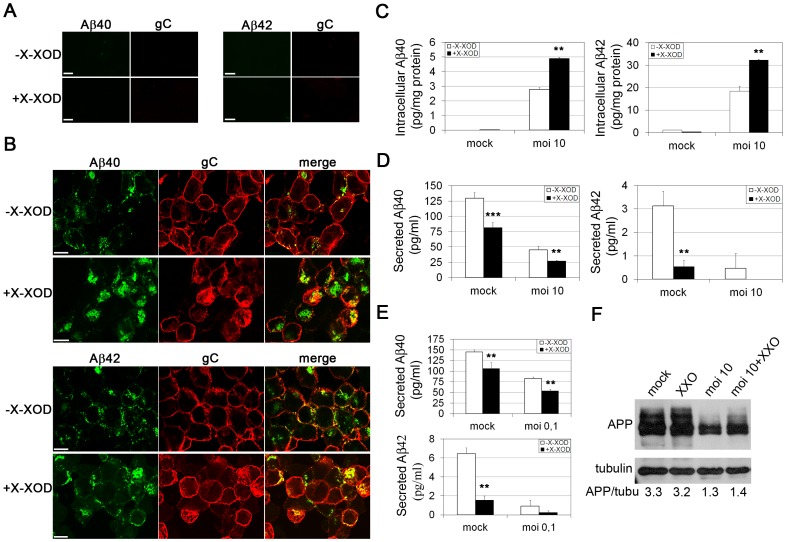
Effects of oxidative stress on Aβ levels in HSV-1-infected SK-APP cells. **A)** Confocal microscopy images showing Aβ40, Aβ42 and gC immunoreactivities in SK-APP cells non-treated or treated with X-XOD for 18 h. Scale bar: 10 µm. **B)** Confocal microscopy images showing Aβ40 and Aβ42 immunoreactivities in cells infected with HSV-1 at a moi of 10 for 18 h (identified with an antibody that recognizes the glycoprotein C of HSV-1) in the presence and absence of X-XOD. Green (Aβ) and red (gC) channels were merged. Scale bar: 10 µm. **C)** ELISA analysis of intracellular Aβ levels, normalized by the amount of total protein, in HSV-1-infected cells at a moi of 10 for 18 h in the presence of X-XOD (**p<0.01). **D and E)** ELISA analysis of Aβ levels in the medium of cells treated with X-XOD and infected with HSV-1 at a moi of 10 for 18 h (**D**) or 0.1 for 42 h (**E**) (**p<0.01; ***p<0.001). In (**C-E**) the results are the means of at least four experiments. Bars show the SEM. **F)** Analysis of APP levels by Western blotting in SK-N-MC cells infected with HSV-1 at a moi of 10 for 18 h. The effect of X-XOD is shown. A control ensuring equal loading - a tubulin blot - is also shown. The ratio of APP to tubulin is shown below the blots. The blots are representative of four independent experiments.

Aβ levels in the SK-N-MC cell line overexpressing the human APP protein (SK-APP) were examined by double labelling immunofluorescence assays. Intracellular Aβ was undetectable in non-infected cells and in cells treated with X-XOD alone (Fig. 1A). In contrast, a strong accumulation of both Aβ species occurred in HSV-1-infected cells in the presence and absence of X-XOD, although Aβ immunoreactivity was stronger in the X-XOD treated cells (Fig. 1B). To quantify the effects of oxidative stress on Aβ production, ELISA assays with antibodies specific for Aβ40 and Aβ42 were performed. As suggested by the immunofluorescence experiments, treatment with X-XOD was unable to induce any increase in intracellular Aβ levels in non-infected cells, but in HSV-1-infected cells it significantly enhanced the cell content of both Aβ isoforms (by about two-fold; Fig. 1C). X-XOD provoked a marked suppression of secretion of Aβ40 (two fold; p<0.001) and Aβ42 (six fold; p<0.01) to the extracellular medium, although in both cases this reduction was smaller than that induced by HSV-1. In addition, X-XOD potentiated the inhibition of Aβ secretion in HSV-1-infected cells, reducing to half the amount of Aβ40 secreted, and Aβ42 to undetectable levels (Fig. 1D). Similar results were obtained when SK-APP cells were infected at a lower dose (moi 0.1 pfu/cell for 48 h), a situation more like that of natural HSV-1 infection (Fig. 1E). X-XOD did not affect the amount of APP either in mock- or HSV-1-infected cells, ruling out the possibility that the modification of Aβ levels induced by oxidative stress was due to an alteration in APP content (Fig. 1F).

**Figure 2:**
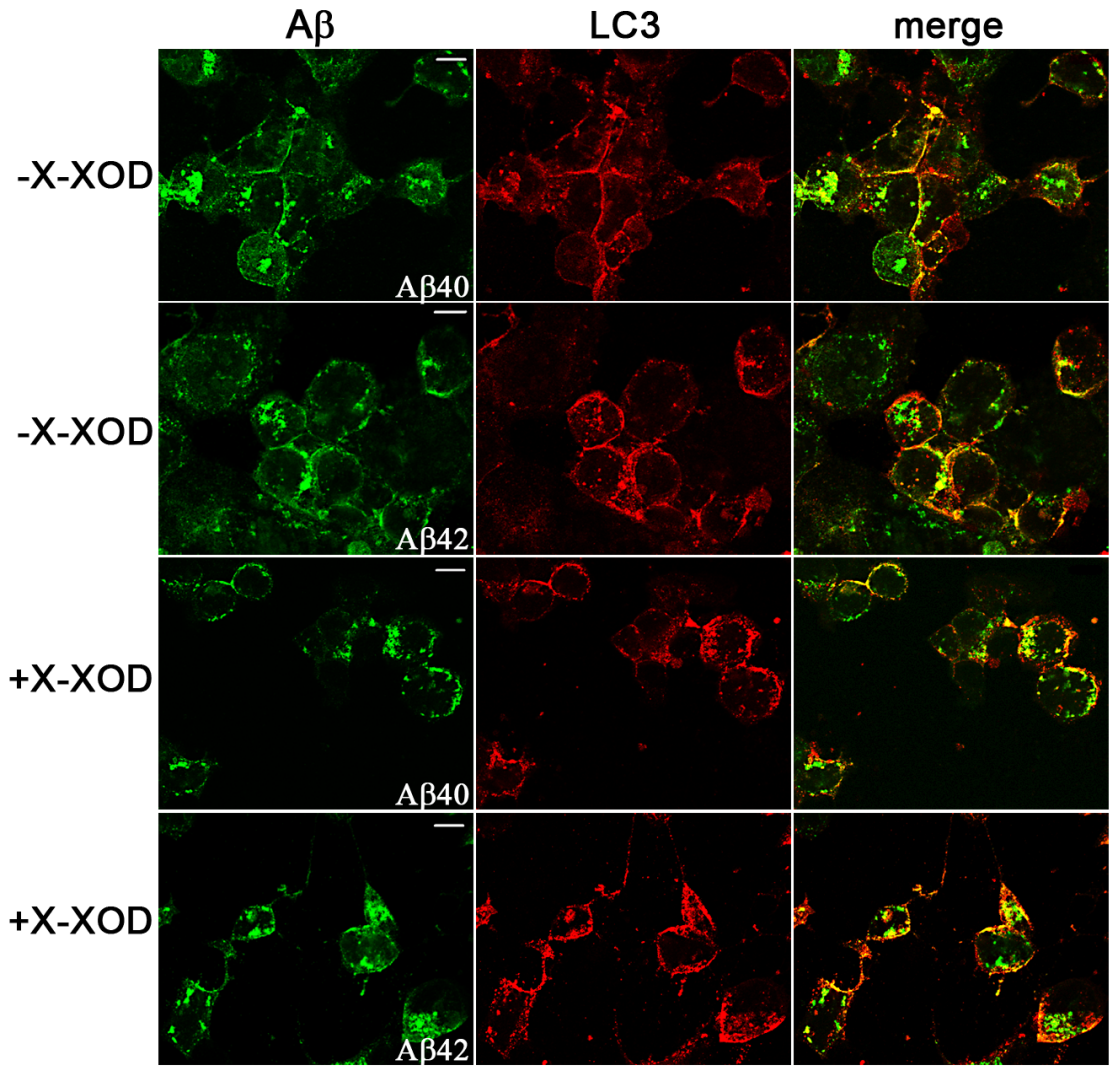
Oxidative stress does not affect the autophagosomal localization of Aβ in HSV-1-infected cells. Confocal images obtained with anti-Aβ and anti-LC3 antibodies showing endogenous LC3 and Aβ patterns in HSV-1-infected SK-APP cells at a moi of 10 for 18 h in the presence and absence of oxidative stress (X-XOD). Colocalization is shown by yellow fluorescence signals in the merged panels. Scale bar: 10 µm.

We previously described Aβ to accumulate in autophagic compartments in HSV-1-infected cells [5]. To determine whether oxidative stress affected this accumulation, the distribution of endogenous LC3, an autophagic vesicle marker, and Aβ was investigated in its presence. Confocal microscopy analysis revealed immunoreactive Aβ structures to colocalize with endogenous LC3-positive vesicles in HSV-1-infected cells independent of their oxidative status (Fig. 2). In contrast, Aβ and LC3 immunoreactivities were undetectable in mock-infected cells (data not shown). Altogether, these results suggest that oxidative stress significantly increases the accumulation of intracellular Aβ and the inhibition of its secretion (both of which are caused by HSV-1 infection) but does not alter the accumulation of Aβ in autophagosomes in HSV-1-infected cells.

### Oxidative Stress Enhances the Autophagosome Accumulation Induced by HSV-1 Infection

**Figure 3:**
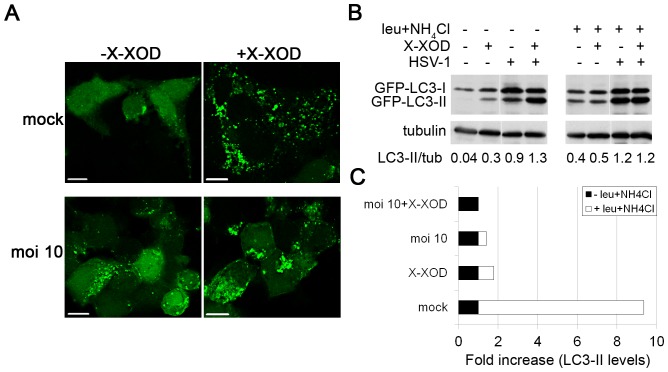
Effects of oxidative stress on autophagy. **A)** SK-LC3 cells were simultaneously treated with X-XOD and infected with HSV-1 at a moi of 10 for 18 h and the cellular distribution of GFP-LC3 assessed by fluorescence microscopy. Scale bar: 10 µm. **B)** Analysis of GFP-LC3 levels by Western blotting with an anti-GFP antibody in SK-LC3 cells simultaneously treated with X-XOD and infected with HSV-1 (moi of 10 for 18 h). The effects of the lysosomal inhibitors leupeptin and ammonium chloride are shown. Blots are representative of four independent experiments; a tubulin blot was performed as a loading control. The ratio of LC3-II to tubulin is shown below the blots. **C)** The graph represents the fold increase in GFP-LC3-II levels, normalized by tubulin, in leupeptin/NH_4_Cl-treated cells compared to non-treated cells in all conditions assayed in (**B**).

LC3 is the most widely monitored autophagy-related protein. LC3 is a ubiquitin-like protein initially synthesized in an unprocessed form, proLC3, which is converted into a proteolytically processed form lacking amino acids from the C-terminus termed LC3-I. LC3-I can be conjugated to phosphatidylethanolamine upon the activation of autophagy, leading to the formation of the isoform LC3-II that remains bound to double membrane autophagic vesicles [25]. We previously reported HSV-1 to induce the accumulation of autophagosomes in a viral dose- and time-dependent manner [12]. To determine whether oxidative stress affected this, the distribution of GFP-LC3 in SK-N-MC cells infected with HSV-1 and stably expressing the GFP-LC3 fusion protein (SK-LC3) was monitored in the presence of X-XOD (Fig. 3A). Fluorescence microscopy analysis showed a cytosolic distribution but few GFP-LC3 spots were seen in non-treated, non-infected cells. In contrast, in X-XOD-treated or infected cells, a strong increase was seen in the number of cells with numerous GFP-LC3 spots. Certainly, the accumulation of GFP-LC3 spots appeared greater in HSV-1-infected cells treated with X-XOD than in non-treated infected cells (Fig. 3A).

To better quantify the effects of oxidative stress, GFP-LC3-II levels were analysed by immunoblotting (Fig. 3B). Consistent with the data obtained in the fluorescence microscopy experiments, increased levels of GFP-LC3-II were observed in SK-LC3 cells following the addition of X-XOD. These results agree with a recent report from our group showing an increase in endogenous LC3-II in SK-N-MC cells treated with X-XOD [31], and confirm that oxidative stress induces autophagosome accumulation in human neuroblastoma cells. HSV-1 infection also induced a strong increase of GFP-LC3-II levels. A marked increase in GFP-LC3-II following X-XOD addition was also observed in HSV-1-infected cells by densitometric analysis of the GFP-LC3 bands (Fig. 3B). These data indicate that oxidative stress not only induces autophagosome accumulation but enhances the autophagosome accumulation induced by HSV-1.

### Oxidative Stress Enhances the Inhibition of the Autophagic Flux Provoked by HSV-1

The accumulation of autophagosomes at determined time points results from either an increase in the rate of their formation or a reduction in autophagosome turnover. To investigate the cause of this accumulation in cells subjected to oxidative stress, a series of experiments was performed to measure the autophagic flux. Autophagic flux refers to the entire process of autophagy, including the delivery of cargos to lysosomes, their subsequent breakdown, and the release of the resulting macromolecules to the cytosol [33]. The effect of oxidative stress on LC3-II turnover in the presence of the lysosomal inhibitors leupeptin and ammonium chloride was determined by immunoblotting (Fig. 3B). A strong increase in GFP-LC3-II levels (∼9-fold) was observed in mock-infected cells in the presence of lysosomal inhibitors, indicating the high level of autophagic flux in SK-LC3 cells. In contrast, the addition of X-XOD only caused a modest increase in GFP-LC3-II levels (∼1.6-fold) (Fig. 3C). We recently reported similar results when the effect of oxidative stress on endogenous LC3 levels was examined in the presence of lysosomal inhibitors [31]. These inhibitors induced a smaller increase in GFP-LC3-II levels in HSV-1-infected cells (∼1.4-fold). The increase induced by lysosomal inhibitors in cells treated with X-XOD, or infected with HSV-1, was completely abolished when both stimuli were combined (Fig. 3C). Taken together, these results suggest that autophagy is induced as a consequence of oxidative stress and HSV-1 infection, but that the final stages of autophagy are inhibited.

**Figure 4:**
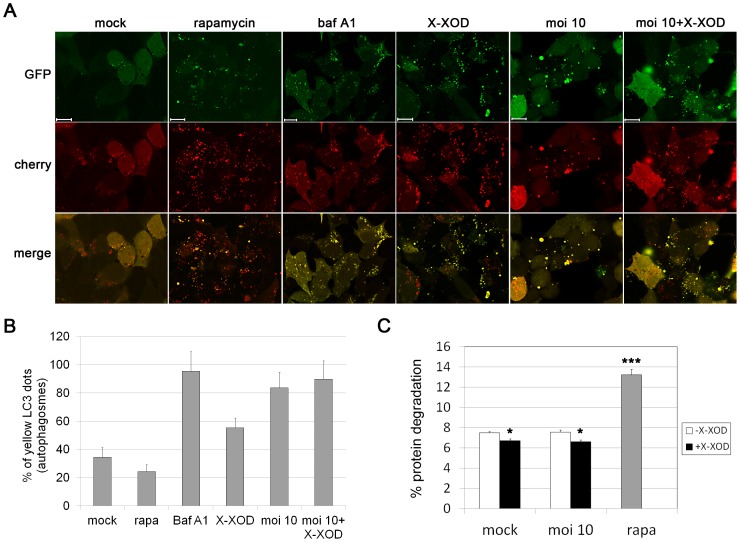
Oxidative stress and HSV-1 infection induced inefficient fusion between autophagosomes and lysosomes. **A)** SK-N-MC cells were transfected with dtLC3 and then infected with HSV-1 at a moi of 10 for 18 h, or treated with bafilomycin A1 (baf A1), rapamycin (rapa) or X-XOD for 18 h. Representative confocal microscopy images are shown; the GFP (green) and mCherry (red) channels were merged. Scale bars: 10 µm. **B)** Graphic representation of the proportion of autophagosomes (yellow dots). The number of fluorescent bodies in 100 cells was counted for each condition. **C)** Degradation of long-lived proteins in SK-N-MC cells infected with HSV-1 at a moi of 10 for 18 h in the presence or absence of X-XOD. Rapamycin (rapa) was used as a positive control of autophagy stimulation. Results are the mean ± SEM of three independent experiments performed in triplicate (*p<0.05; ***p<0.001).

The accumulation of LC3-II can be obtained by interrupting the autophagosome-lysosome fusion step. To determine whether this is impaired by oxidative stress, SK-N-MC cells were transfected with a tandem repeat GFP-mCherry-LC3 construct [26]. This reporter shows dual red-green fluorescence in phagophores and autophagosomes, but loses the GFP signal in the acidic environment of autolysosomes. X-XOD treatment increased the appearance of both GFP-LC3 and mCherry-LC3 fluorescent spots and their colocalization (“yellow” vesicles, indicative of autophagosomes) compared to that seen in non-treated cells. A significant increase in the number of yellow-fluorescence vesicles in HSV-1-infected cells was also seen, confirming previous results [12]. In contrast, control and rapamycin-treated cells demonstrated functional autophagy with a preponderance of autolysosomes (“red-only” LC3 vesicles) (Fig. 4A). These data suggest that X-XOD blocks the autophagic flux. To quantify the inhibition of autophagosome-lysosome fusion, the proportion of autophagosomes (“yellow” vesicles) was calculated. This proportion increased greatly in cells exposed to X-XOD (58%) or HSV-1 (80%) compared to control and rapamycin-treated cells (35% and 24% respectively). It further increased to 90% when the cells were treated with both X-XOD and HSV-1, although this increase was not statistically significant. This ratio was similar to that obtained in cells treated with bafilomycin A1, an inhibitor of autophagosome-lysosome fusion (Fig. 4B).

Finally, the effect of oxidative stress on autophagic protein degradation was tested using long-lived protein degradation assay, a well established method for measuring autophagic flux [33]. As previously reported, HSV-1 did not affect the long-lived protein degradation rate [12]. Rapamycin, a classical inducer of autophagy, significantly increased the degradation of long-lived cell proteins as expected. In contrast, X-XOD treatment resulted in a significant reduction of protein degradation, both in mock- and HSV-1-infected cells (Fig. 4C).

These results suggest that oxidative stress provokes inefficient fusion between autophagosomes and lysosomes, resulting in reduced autophagic protein degradation and potentiation of the impairment of autophagy induced by HSV-1 infection.

### Oxidative Stress Inhibits HSV-1 Replication

The additive effects of oxidative stress on neurodegeneration events induced by HSV-1 could be argued due to the facilitation of infection. To test this hypothesis, the levels of several viral proteins, viral DNA and infectious particles were monitored in the presence of the X-XOD system.

**Figure 5:**
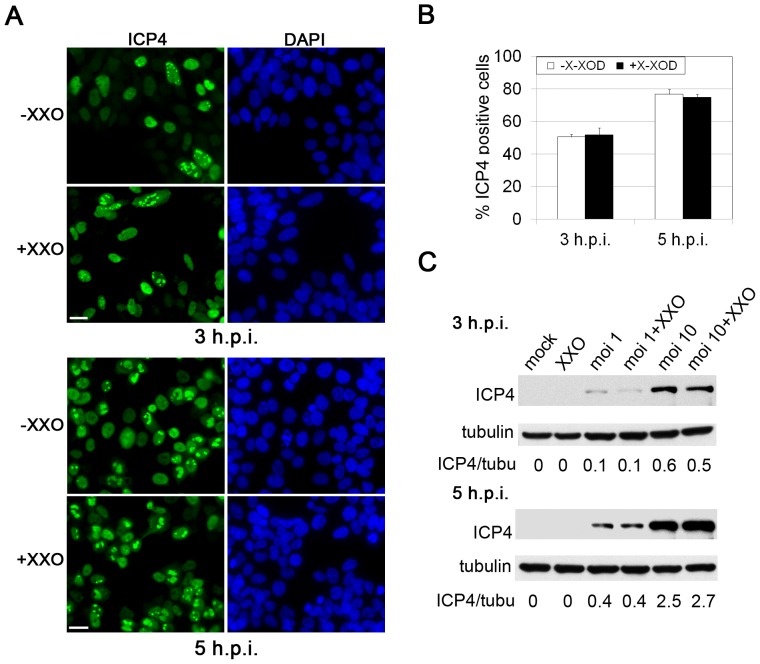
Effects of oxidative stress on HSV-1 entry. **A)** Immunofluorescence analysis of HSV-1-infected SK-N-MC cells at a moi of 10 in the presence and absence of X-XOD. The immunoreactivity of ICP4 protein is shown at 3 and 5 hours post-infection (h.p.i.). Nuclei are stained with DAPI. Scale bar: 20 µm. **B)** Quantification of infected cells by ICP4 staining. The graph shows the percentage of ICP4-positive cells. At least 400 nuclei were counted for each condition. **C)** Analysis of ICP4 levels by Western blotting in SK-N-MC cells infected with HSV-1 at a moi of 1 and 10 for 3 h and 5 h, in the presence and absence of X-XOD.

To determine whether oxidative stress enhances viral entry into SK-N-MC cells, the latter were simultaneously treated with X-XOD and infected with HSV-1 and the number of infected cells at early stages of infection (3 and 5 h.p.i. [hours post-infection]) determined by immunofluorescence analysis using a specific antibody for ICP4 (Fig. 5A). ICP4 is coded for by an immediate-early gene, the expression of which begins at early stages of infection, and it accumulates in the nucleus of infected cells. The quantification of ICP4-positive cells revealed the percentage of infected cells not to be modified by X-XOD treatment (Fig. 5B). Consistent with these results, immunoblotting showed the ICP4 levels to remain constant in HSV-1-infected cells in the presence of X-XOD (Fig. 5C). These data indicate that oxidative stress does not facilitate viral entry into human neuroblastoma cells.

**Figure 6:**
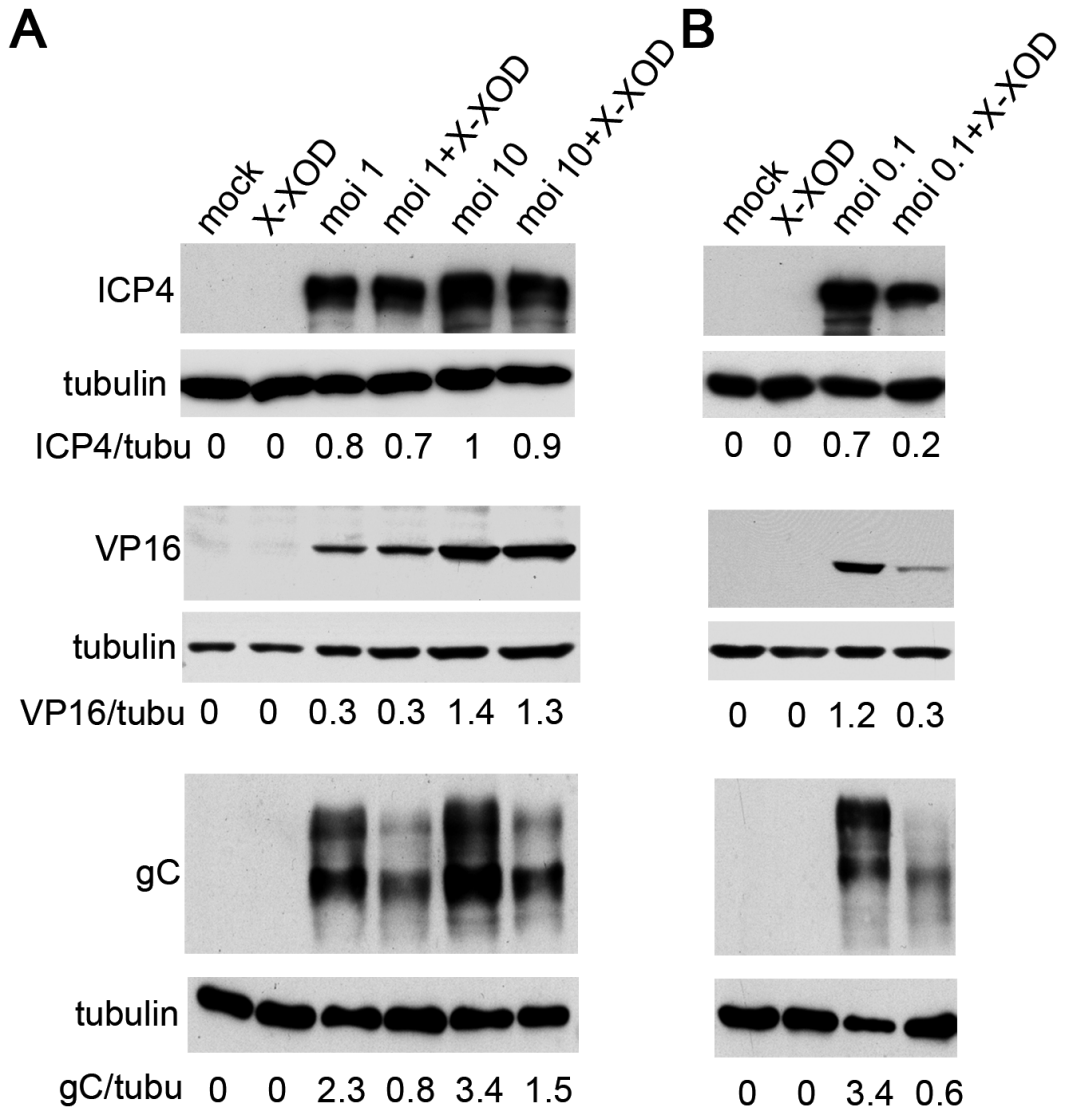
Effects of oxidative stress on HSV-1 protein expression. The accumulation of viral proteins ICP4, VP16 and gC was analysed by immunoblotting in SK-N-MC cells simultaneously treated with X-XOD and infected with HSV-1 at a moi of 1 and 10 for 18 h (**A**) or at a moi of 0.1 for 42 h (**B**). The blots shown are representative of three independent experiments. A tubulin blot was performed as a loading control. In all blots, the ratio of viral proteins to tubulin is shown below the blots.

**Figure 7:**
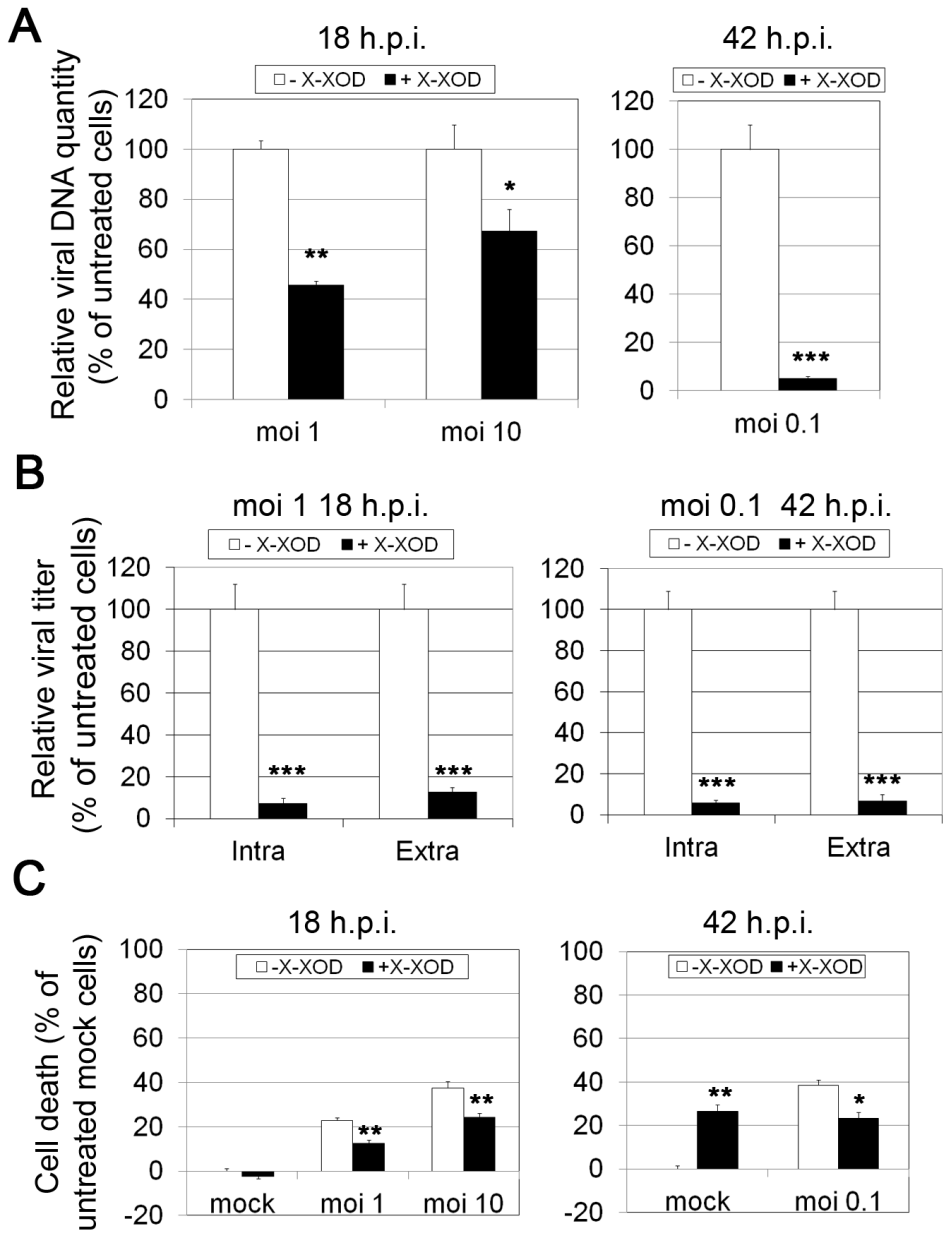
Oxidative stress reduces HSV-1 replication and increases cell viability of infected cells. **A)** Quantification of viral DNA by real-time quantitative PCR in SK-N-MC cells simultaneously treated with X-XOD and infected with HSV-1 at a moi of 1 and 10 for 18 h or at a moi of 0.1 for 42 h. **B)** Intracellular (intra) and extracellular (extra) viral titres were determined by plaque assays in SK-N-MC cells infected under the same conditions as in (**A**). In (**A**) and (**B**), the data represent the mean ± SEM of five experiments performed in triplicate and are expressed as a percentage with respect to untreated cells (- X-XOD) (*p<0.05; **p<0.01; ***p<0.001). **C)** The cell viability of mock and HSV-1-infected SK-N-MC cells exposed to X-XOD was monitored using the MTT reduction assay. Cells were infected with HSV-1 at a moi of 1 and 10 for 18 h or at a moi of 0.1 for 42 h. Values are expressed relative to the optical density of untreated mock-infected cells. The data shown represent the mean ± SEM for four independent experiments performed in triplicate (*p<0.05; **p<0.01).

To determine whether oxidative stress has any effect on viral protein synthesis, the expression of representative viral proteins at late stages of infection (18 h.p.i.) was assessed in SK-N-MC cells in the presence of X-XOD. Immunoblotting analyses were performed using antibodies specific for ICP4 (an immediate early protein, the expression of which begins before HSV-1 DNA replication takes place), VP16 (a γ_1_ late protein, the expression of which is not strictly dependent on viral DNA synthesis) and glycoprotein C (gC, which belongs to the class of γ_2_ “true late” genes, the expression of which requires viral DNA synthesis). When SK-N-MC cells were infected with different viral doses (moi 1 and 10) in the presence of X-XOD, no differences in the amount of ICP4 were observed compared to non-treated infected cells at 18 h.p.i. (Fig. 6A). Similar levels of VP16 were detected in HSV-1-infected cells irrespective of the addition of X-XOD (Fig. 6A). Finally, X-XOD treatment greatly reduced gC levels in infected cells (67% of reduction at a moi of 1 and 55% at a moi of 10; Fig. 6A), indicating that oxidative stress only altered the levels of viral proteins whose expression is strictly dependent on viral DNA synthesis. When the levels of HSV-1 proteins were assessed in SK-N-MC cells infected for longer, i.e., when a replication cycle had been completed (moi 0.1 for 42 h), large reductions in ICP4, VP16 and gC levels were recorded (Fig. 6B). These results indicate that oxidative stress reduces viral protein levels in the second replication cycle. It may therefore inhibit the replication of viral DNA and protect from HSV-1-induced cell death. To test this hypothesis, viral DNA replication was analysed in real-time quantitative PCR assays (Fig. 7A). X-XOD caused a significant reduction in viral DNA levels under the different moi conditions assayed (a 55% and 33% reduction at moi 1 and 10 respectively at 18 h.p.i, and >95% at moi 0.1 at 42 h.p.i). Finally, the effect of oxidative stress on HSV-1 titres was measured by plaque assays. A strong reduction in the production of infectious HSV-1 particles was seen after X-XOD addition (>90% in all conditions assayed; Fig. 7B). Since oxidative stress induced the inhibition of HSV-1 replication, its effect on the viability of HSV-1-infected cells was also tested. We have previously reported that the oxidative stress induced by the X-XOD treatment leads to apoptotic cell death in human neuroblastoma cells, starting at 36 h [34]. In the present work, MTT assays revealed significantly reduced cell viability after 42 h of exposure. At 18 h.p.i., the number of dead cells increased with viral dose, reaching ∼ 40% at a moi of 10 (Fig. 7C). When the SK-N-MC cells were infected at a moi 0.1 for 48 h, a situation more like that of natural HSV-1 infection, the proportion of dead cells reached 40%. Treatment with X-XOD led to a significant reduction in cell death in HSV-1-infected cells at all viral doses and times assayed (Fig. 7C).

Taken together, these results confirm that oxidative stress reduces HSV-1 replication and protects against cell death provoked by the virus in human neuroblastoma cells.

## Discussion

Viral infections are commonly associated with the appearance of oxidative stress in infected cells. Certainly, HSV-1 shifts the intracellular redox balance towards a pro-oxidant state (reviewed in [23], [24]). Oxidative damage could therefore be an infection-induced mechanism of neuronal injury. However, the interplay between oxidative stress and HSV-1 infection has not been extensively studied and, until now, it was unknown whether these two factors interact in some way to promote neurodegeneration.

The free radical-generating X-XOD system has been employed in the experimental induction of oxidative stress [35]. In earlier work we developed a human neuronal cell model of mild oxidative stress using this system that allowed the analysis of free radical-induced events preceding cell death. We recently reported the X-XOD system to alter cholesterol biosynthesis [34] and APP metabolism/processing [31], [32]. Since X-XOD-modulated processes are altered in the AD brain, we investigated the effects of X-XOD on AD-like neurodegeneration events induced by HSV-1 infection.

We have previously shown that HSV-1 impairs two processes intimately related to neurodegeneration: autophagy and the APP proteolytic process. HSV-1 induces an abortive autophagic response resulting in autophagosome accumulation [12]. Additionally, the intracellular accumulation of Aβ in autophagic compartments induced by HSV-1 infection is caused by the inhibition of Aβ secretion and the failure of Aβ degradation by autophagy [5]. We therefore performed a series of experiments to examine the relationship between HSV-1 and oxidative stress. In human neuroblastoma cells, oxidative stress was found to significantly enhance the accumulation of intracellular Aβ and the inhibition of Aβ secretion mediated by HSV-1 infection. With respect to autophagy, oxidative stress potentiated the accumulation of autophagic compartments as a result of an increase of the inhibition of autophagic flux in infected cells. Consistent with these data, oxidative stress did not affect Aβ accumulation in autophagic vesicles in infected cells. In earlier work, we showed that mild oxidative stress increases LC3-II and p62 levels, both substrates of autophagic process, suggesting that the observed accumulation of autophagosomes was due to the interruption of autophagic flux [31]. In this report, pH-sensitive double-tagged mCherry-GFP-LC3 assay confirms that oxidative stress induces abnormal autophagy via inefficient fusion between autophagosomes and lysosomes in neuronal cells. This oxidative stress-mediated inhibition of autophagic flux could contribute to the increased accumulation of intracellular Aβ in autophagosomes in HSV-1-infected cells. It has been reported that HSV-1 is able to modulate autophagy through two viral proteins. The HSV-1 neurovirulence protein ICP34.5 blocks autophagy by promoting eIF2α dephosphorylation and by inhibiting the essential autophagy protein beclin 1 [36], [37]. In addition, the late viral protein Us11 is also able to block autophagy by direct inhibition of the double-stranded RNA-dependent protein kinase PKR [38]. These findings reveal that HSV-1 infection strongly controls the autophagic process through multiple mechanisms.

The question arises as to whether the enhancement of neurodegeneration events provoked by oxidative stress is merely due to greater facilitation of infection. However, oxidative stress was seen to have no effect on the number of infected cells at early stages of infection or on the levels of immediate early (α) and leaky late (γ1) proteins. This indicates that oxidative stress does not affect virus entry or the transcription of replication-independent genes. In addition, a significant reduction in true late protein (γ2) gC levels was observed. The expression of true late γ2 class genes strictly depends on viral DNA synthesis. This suggests that the viral DNA production is affected by oxidative stress. Indeed, analysis of HSV-1 DNA replication showed a significant reduction in viral DNA levels and an inhibition of the formation of new infectious particles induced by oxidative stress. Consistent with our results, the cellular antioxidant chaperone Hsp27 is required for efficient HSV-1 replication [39] and the accumulation of oxidized proteins was enhanced in infected cells in the absence of Hsp27 [40]. However, the inhibition of the formation of new infectious particles in the presence of oxidative stress was much stronger than the inhibition of viral DNA replication, suggesting that an additional defect in a late stage of infection might occur (e.g. maturation of viral particle). The present results therefore confirm that oxidative stress reduces HSV-1 replication and protects human neuroblastoma cells from death provoked by HSV-1 infection. In addition, they show that the strengthening of neurodegeneration markers induced by oxidative stress in HSV-1-infected cells cannot be attributed to any facilitation of infection. Further investigations are needed to better understand the basic mechanisms involved in these alterations. In this respect, we are currently engaging in microarray analyses of differential gene expression in cellular infection models in order to identify the genes and/or pathways involved in the effects of oxidative stress on HSV-1 infection.

Few reports exist on the effects of oxidative agents on HSV-1 infection. In agreement with the present results, a recent report showed that HSV-1 contains catalase, an enzyme that catalyses the decomposition of hydrogen peroxide. This suggests viral replication needs to be protected from oxidising environments [41]. In contrast, Arimoto et al. showed hydrogen peroxide to promote the release of HSV-1 from epithelial cells, increasing the amount of cell-free virus without reducing levels of cell-associated virus [42]. The difference between the present results and those obtained by Arimoto et al. might be explained in several ways: i) the use of different cell types, ii) maintaining the oxidation inducer throughout the experiment in the present work, but adding it just 2 h before the end of infection in the work of these other authors, and iii) the intensity of the oxidative damage generated. Hydrogen peroxide at the concentration used in the Arimoto study (1 and 5 mM) acts as a strong oxidizing agent that causes a significant increase in cell death just 2 h after its addition, whereas the X-XOD system used in the present work induces mild oxidative stress that does not affect cell viability at 18 h.

Since oxidative stress blocks HSV-1 replication, a reduction in the neurodegenerative effects induced by infection might be expected. However, the accumulation of autophagosomes and intracellular Aβ observed in infected cells treated with X-XOD was significantly stronger than in infected but non-X-XOD-treated cells. Consistent with this finding, previous experiments performed in human neuronal cells in the presence of the viral DNA synthesis inhibitor phosphonoacetic acid showed that a productive infection is not required to induce all the AD-like neurodegeneration events [5], [7], [12]. Since the non-treated cells were more susceptible to HSV-1 replication than were the X-XOD-treated cells, the rapid lysis of the former may have precluded stronger Aβ and autophagosome production. In this scenario, oxidative stress might attenuate the infection process, maintaining the negative effects of infection on the host cells for longer.

Another possibility is that oxidative damage provides a second mechanism by which HSV-1-induced neuronal injury may occur. Consequently, oxidative stress produced *in vitro* (X-XOD treatment) or *in vivo* (microglial response) may enhance the neurodegenerative effects provoked by HSV-1. In line with this hypothesis, De Chiara et al. [3] found that antioxidant compounds prevented the formation of Aβ oligomers induced by HSV-1 in SH-SY5Y human neuroblastoma cells, suggesting that oxidative stress is involved in this process. In addition, alterations in the redox state have been associated with the appearance of the characteristic pathological anomalies that accumulate in AD brains. Indeed, numerous studies have shown that pro-oxidant agents can increase the production of Aβ [43], [44], and a recent report has revealed that antioxidant therapy interrupts the progression of amyloid and tau pathology in a mouse model of AD [45]. Further, several kinases involved in tau phosphorylation belong to the stress-activated protein kinase family known to be activated during oxidative stress [46].

In summary, the present findings indicate that, in the present model, mild oxidative stress caused by the X-XOD system strongly affects the neurodegeneration markers induced by HSV-1; it significantly enhances the effects of HSV-1 on Aβ accumulation and secretion and contributes to the impairment of autophagy observed in HSV-1-infected cells (thus increasing the inhibition of autophagic flux). Interestingly, HSV-1 replication is severely compromised in cells subjected to oxidative stress; the effects of oxidative stress on neurodegeneration events cannot, therefore, be a consequence of an increase in HSV-1 replication. Taken together, the present results identify an interesting link between oxidative stress and HSV-1 infection; their interaction may promote neurodegeneration events in AD brains.
